# Comprehensive fluid dynamic quantification in congenital heart disease: introduction of a new software tool

**DOI:** 10.1186/1532-429X-17-S1-P70

**Published:** 2015-02-03

**Authors:** Dominik Daniel Gabbert, Christopher Hart, Inga Voges, Philip Wegner, Michael Jerosch-Herold, Ines Kristo, Traudel Hansen, Hans-Heiner Kramer, Carsten Rickers

**Affiliations:** 1 Deptartment of Congenital Heart Disease and Pediatric Cardiology, UKSH Kiel, Kiel, Germany

## Background

Recent independent approaches have been presented to describe and quantify particular properties of blood flow using MRI. However, the fluid dynamic data acquired with 4D phase contrast involve complex flow patterns that motivate a systematic, comprehensive analysis of various quantities in a single software tool. For the hemodynamics, an undisturbed vascular forward flow is crucial. In this context, the evaluation of power loss due to turbulences and the study of flow vortices and their impact are important contributions to gain deeper insight into cardiovascular flow.

## Methods

We developed a prototype of a software tool that allows a comprehensive analysis of flow quantities such as velocity, vorticity, helicity and turbulent kinetic energy. The identification of vessels is achieved by applying a threshold-based segmentation on a contrast-enhanced MR angiography scan. Cross-sectional planes are defined and angulated by the user in order to obtain vessel cross-sections and the intermediate vessel volumes between such planes. Velocity, vorticity, helicity and turbulent kinetic energy are calculated voxel-wise in each time frame from 4D PC data. The quantities are then averaged over volume for each time frame. The software is developed on the MeVisLab platform. It was tested on three patients.

## Results

The calculations of the desired quantities were performed successfully for all three cases. An example for results of vorticity are visualized in the figure. Numerical results for all quantities are given in the table.

**Figure 1:**
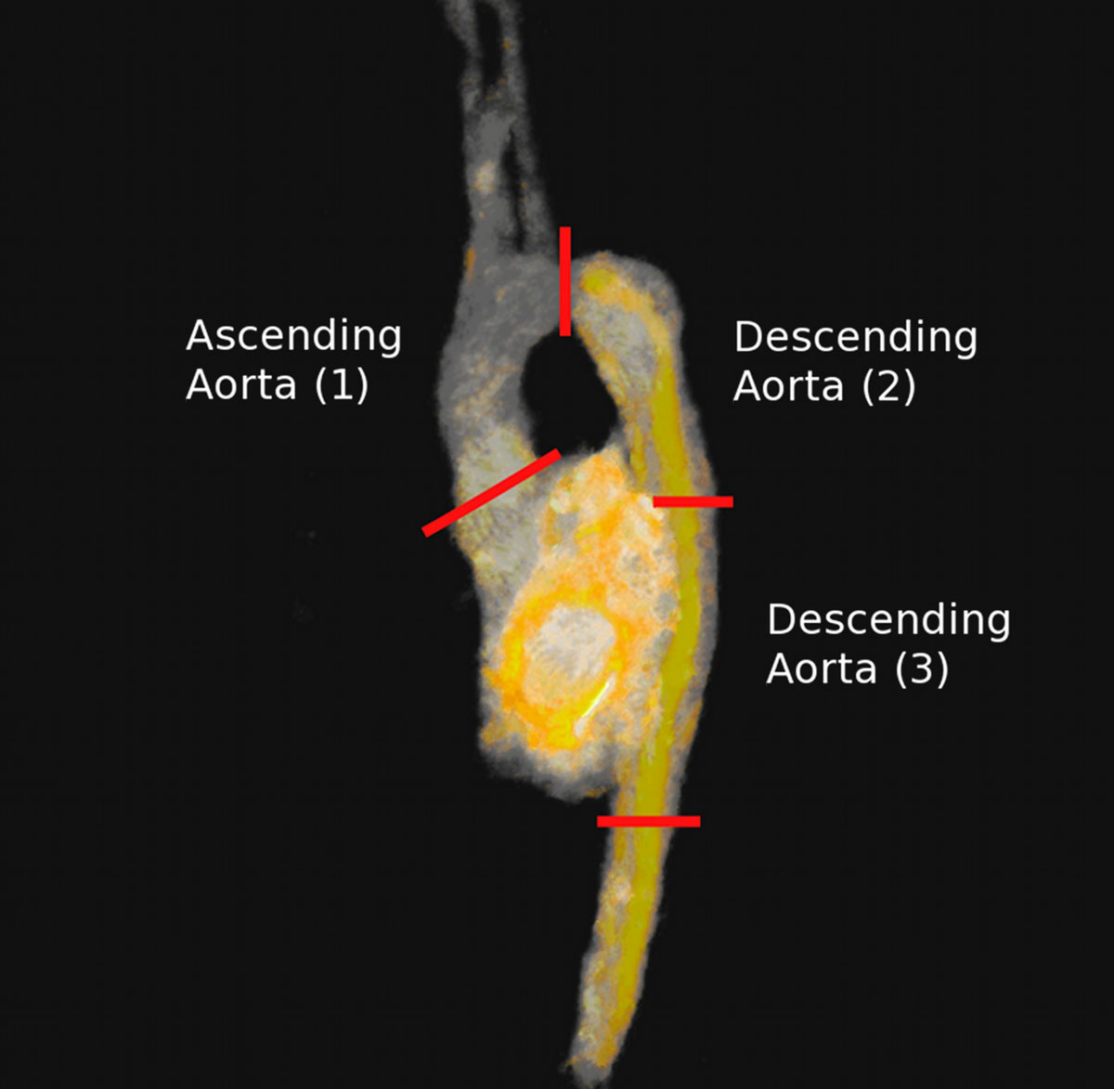
Visualization of vorticity results in the aorta. Three segments are defined as used in this analysis.

**Table 1:** Results for values of velocity, vorticity, relative helicity and turbulent kinetic energy averaged over the corresponding segment volume for three patients with aortic coarctation. The results in the table are maximum values reached within the cardiac cycle.

	Velocity [cm/s]	Vorticity [1/s]	Relative Helicity	TKE [J/m3]
Segment 1	105 ± 31	8 ± 3	0.09 ± 0.12	189 ± 29

Segment 2	96 ± 27	14 ± 5	0.23 ± 0.15	310 ± 68

Segment 3	82 ± 33	10 ± 3	0.31 ± 0.09	224 ± 43

## Conclusions

A comprehensive analysis of complex flow quantities can be accomplished by a single software. The method is general enough for new algorithms to be developed and implemented. We intend to apply the analysis software to specific needs in congenital heart disease in order to obtain a global assessment of the fluid dynamic situation.

## Funding

N/A.

